# The burden of headache disorders in Ethiopia: national estimates from a population-based door-to-door survey

**DOI:** 10.1186/s10194-017-0765-7

**Published:** 2017-05-25

**Authors:** Mehila Zebenigus, Redda Tekle-Haimanot, Dawit K Worku, Hallie Thomas, Timothy J Steiner

**Affiliations:** 1 Department of Neurology, School of Medicine, College of Health Science, Addis Ababa University, Addis Ababa, Ethiopia; 2 Department of Internal Medicine, School of Medicine, College of Health Science, Addis Ababa University, Addis Ababa, Ethiopia; 3 Department of Internal Medicine, Bahir Dar University, Bahir Dar, Ethiopia; 4 Department of Neuromedicine and Movement Science, Faculty of Medicine and Health Sciences, NTNU Norwegian University of Science and Technology, Edvard Griegs Gate, NO-7491 Trondheim, Norway; 5 Division of Brain Sciences, Imperial College London, London, UK

**Keywords:** Epidemiology, Burden of disease, Headache, Migraine, Tension-type headache, Medication-overuse headache, Ethiopia, Sub-Saharan Africa, Global campaign against headache

## Abstract

**Background:**

Headache disorders are the third-highest cause of disability worldwide, with migraine and medication-overuse headache (MOH) the major contributors. In Ethiopia we have shown these disorders to be highly prevalent: migraine 17.7%, TTH 20.6%, probable MOH (pMOH) 0.7%, any headache yesterday (HY) 6.4%. To inform local health policy, we now estimate disability and other burdens attributable to headache in this country.

**Methods:**

In a cross-sectional survey using cluster-randomized sampling, we visited households unannounced in four diverse regions (urban and rural) of Ethiopia. We interviewed one member (18–65 years old) of each household using the HARDSHIP structured questionnaire. Screening and diagnostic questions based on ICHD-II were followed by burden enquiry in multiple domains. We estimated disability using disability weights (DWs) from the Global Burden of Disease 2013 study.

**Results:**

We interviewed 2385 participants (1064 [44.7%] male, 596 [25.0%] urban; participating proportion 99.8%). Reported mean intensity of migraine was 2.6 (scale 1–3). People with migraine spent 11.7% of their time in the ictal state (DW: 0.441); they were therefore 5.2% disabled overall. Pain and disability from TTH were much lower. Mean intensity of pMOH was 2.95. People with pMOH spent 60.2% of time with headache (DW: 0.223), and were 13.4% disabled. Average proportions of per-person lost productive time were, for migraine, 4.5% from paid work, 5.3% from household work; for pMOH they were 29.2% and 16.0%. There were highly-disabled minorities, and large gender differences, males losing more paid workdays, females more household workdays. All headache types were associated with impairments in quality of life. Across the population aged 18–65 years (effectively the working population), disability from headache was 1.4%, with 1.6% of workdays lost (half from migraine). Estimates from HY, eliminating recall error, were highly compatible.

**Conclusions:**

Ethiopia is a low-income country, and cannot afford these losses – including, perhaps, 1.6% of GDP. Political action is necessary, aimed at mitigating both the economic burden and the associated ill health. WHO has recommended structured headache services with their basis in primary care as the most efficient, effective, affordable and equitable solution, potentially cost-saving. We believe they can be implemented within Ethiopia’s existing health-care infrastructure.

## Background

The Global Campaign against Headache is conducted by *Lifting The Burden* (LTB), a UK-registered non-governmental organization in official relations with the World Health Organization (WHO) [1]. Its ultimate purpose is to reduce the burden of headache worldwide [2–4]. At its launch in 2004, our knowledge of both the scope and scale of the burdens attributable to headache was extraordinarily imprecise; from very large areas of the world, there were almost no reliable data [5]. In the 13 years since, LTB has endeavoured to fill these knowledge gaps, undertaking or supporting a series of population-based studies in countries around the world [6–13] using standard methodology and survey instruments [14, 15]. Findings from these studies have informed various iterations of the Global Burden of Disease (GBD) study [16–18], while providing country policy-makers with local knowledge to guide priority-setting in health care.

In this process, headache disorders have been revealed as the third-highest cause of disability worldwide [17, 19]. Migraine and medication-overuse headache (MOH) are the major contributors [18, 19], being highly disabling at individual level; tension-type headache (TTH), while less disabling at this level, is the second most prevalent disorder in the world [17]. All three of these disorders are therefore important causes of public ill health globally. They place heavy demands on health services, and call upon policy-makers in control of health-resource allocation to give them due attention [19–21]. Worldwide, however, policy-makers have notably failed in the last regard, a situation to which WHO has not only called attention but also urged action to provide remedy [21].

It is unquestionably challenging to implement health-care solutions for the very large numbers of people affected by these disorders. On the other hand, equally unquestionably, it is not humane to ignore the burdens of ill health and disability that they impose. Neither is it economically sensible: effective treatments exist [22] and, appropriately used, they are likely to be cost-saving in most economies [21, 23]. Why does nothing happen [19, 24]?

There is no clear answer to this question, but more persuasive data appear to be needed at national level; therefore, the Global Campaign’s series of country-based studies continues. For Ethiopia, we have already published prevalence data: 44.9% of participants in our population-based survey (males 37.7%, females 49.9%) described headache in the last year, and 7.1% (males 4.1%, females 9.2%) reported headache during the day before enquiry (headache yesterday [HY]) [13]. Adjusted for gender, age and urban or rural habitation, the 1-year prevalence of migraine was 17.7%, of TTH 20.6%, of pMOH 0.7% and of other headache on ≥15 days/month 2.5%. The adjusted 1-day prevalence of any headache was 6.4%.

Headache is therefore very common in this country. Here, expressly as a needs-assessment to inform national health policy, we present data from the same survey on the burdens attributable to headache in Ethiopia. Few studies of headache burden are yet available from sub-Saharan Africa (SSA), but this survey follows, and mirrors, a similar one conducted by LTB in Zambia [10].

## Methods

### Study design and procedures

The methods have been published in detail previously [13]; here they are summarized. During April and May 2014, we conducted a cross-sectional questionnaire-based survey of adult Ethiopian nationals. We sampled from four diverse Regions of Ethiopia, in whole occupied by about 80% of the country’s population: the mostly urban Addis Ababa and its environs, and selected rural districts of Oromia, Amhara and South Nations Nationalities and People’s Regions States (SNNPRS). We used cluster-randomized sampling to select households, called at these households unannounced and randomly selected one member aged 18–65 years of each biologically-unrelated family within each household.

We used a culturally modified version of the structured Headache-Attributed Restriction, Disability, Social Handicap and Impaired Participation (HARDSHIP) questionnaire [15], translated according to LTB’s translation protocol for lay documents [25] into Oromo for the Oromia Region and Amharic for the other three Regions. HARDSHIP included personal and demographic enquiry, and headache screening and diagnostic questions based on ICHD-II [26]. It also included enquiry into various domains of burden: symptom burden (frequency, intensity, duration), lost productive time (from the HALT questionnaire [27], inserted as a module within HARDSHIP), willingness to pay (WTP) for effective health care for headache, if it were available, and quality of life (QoL) (from WHOQoL-8 [28], also inserted as a module). The screening question for headache was: “In the last year, have you had headache?” Participants who answered “yes” were asked if all their headaches were of one or more types and, if more than one, to focus in the subsequent questions on the one that was most bothersome for purposes of diagnosis and burden enquiry. The timeframes for burden enquiry were the preceding 3 months and preceding day, the latter addressed to those responding positively to “Did you have a headache yesterday?”

### Statistics and analysis

We required a minimal sample size of 2000 [13, 14]. Anticipating a 20% non-participation proportion, we aimed to visit 2400 households.

Data from questionnaires were entered into a secure database using double data-entry [13]. Diagnoses were derived algorithmically during analysis [15]. We first separated participants reporting headache on ≥15 days/month, describing these as a separate group. Those among this group who also reported regular use of headache medication on >3 days/week were considered to have probable MOH (pMOH). To all others, the algorithm applied ICHD-II criteria in the order: migraine, TTH, probable migraine, probable TTH [14, 26]. Cases of migraine and probable migraine, and of TTH and probable TTH, were combined for prevalence estimation and burden analyses.

We recorded usual headache intensity on a verbal rating scale in terms meaning “mild”, “moderate” or “severe” (“not bad”, “quite bad” and “very bad”). We transformed these data into a numerical scale 1–3 (0 being no pain), and treated them as continuous. We recorded headache frequency in days affected per month, and usual duration of headache in hours. Both yielded continuous data, which we summarised as means for each headache type. We assumed headache frequency in days per month was equal to attack frequency per month unless reported headache duration was >24 h; when this was the case, we applied a correction factor to avoid over-counting. We derived average time per month spent in the ictal state of each headache type as the product of attack frequency and duration, and expressed it as a proportion of all time (dividing by [30*24]). We calculated headache-attributed disability at individual level as the product of time in ictal state and the disability weight (DW) from GBD2013 [29] for the disorder in question.

We measured lost productivity from HALT in whole days during the preceding 3 months, equating, according to accepted methodology [15, 27], “less than half achieved” to “nothing achieved” and counterbalancing this by equating “more than half” to “everything”. We calculated these losses as proportions of total productive time available. For HY, we reckoned productivity yesterday as either zero (less than half or nothing achieved) or 100% (more than half or everything achieved). We recorded income and WTP in Ethiopian birr (ETB) per month, which we converted to US dollars [USD] at USD 1.00 = ETB 18.93. We assessed QoL as summed scores for the 8 questions of WHOQoL-8 (each 1–5, higher scores indicating better QoL) [28]. These were continuous data, and we summarised them as means ± standard deviations (SDs), medians and quartiles.

Analyses were performed using SPSS/PC version 20.0 software packages for statistical analysis (SPSS, INC, Chicago, IL) and Excel Professional Plus 2010 Version 14.0.7166.5000. We used Student’s t test, chi-squared and Wilcoxon tests to compare distributions and proportions. We regarded *p* < 0.05 as significant.

## Results

From 2528 households approached, 2461 biologically unrelated participants were successfully interviewed; 2385 were included in the analysis (exclusions were aged >65 years). The 61 household refusals were not counted as non-participants since we had not established the presence of anyone eligible [14]. There were six refusals by selected respondents (participating proportion 99.8%).

### Individual burden

We calculated the individual symptom burden arising from each headache type (Table 1).

**Table 1:** Symptom and disability burdens by headache type, at individual and population levels

Burden variable	Migraine (*N* = 452)	Tension-type headache (*N* = 493)	Probable MOH (*N* = 21)	Other headache on ≥15 d/m (*N* = 67)
Mean headache intensity (on scale 0–3)	2.6	2.4	2.95	2.6
Mean headache frequency (attacks/month)	2	1.5	23.3	24.9
Mean duration of attack (hr)	42.2	34.9	18.6	17.9
Mean time in ictal state (% of total time)	11.7	7.3	60.2	61.9
Disability weight (from GBD2013 [29])	0.441	0.037	0.223	0.197^a^
Mean disability per person affected (%)	5.2	0.27	13.4	12.2
Prevalence (adults aged 18–65 year) (%) (from [13])	17.7	20.6	0.7	2.5
Disability in entire 18–65 year-old population (%)	0.92	0.06	0.09	0.30

*MOH* medication-overuse headache, *d/m* days/month, ^a^calculated from DW for probable MOH by adjusting according to relative headache intensities (see text)

For migraine, headache was reported on an average of 3.3 ± 2.6 days/month, with mean intensity of 2.6 ± 0.5 (moderate to severe pain). Duration was highly variable (mean 42.2 ± 84.5 h), with some reports of attacks lasting many days (effectively status migrainosus). As this was >24 h, it implied a mean attack frequency of 2/month, each attack extending into 2 days (Table 1). The proportion of all time spent in the ictal state (calculated as 100*[2*42.2]/[30*24]%) was therefore 11.7%. Disability level (the mean disability attributed to migraine per adult with the disorder), estimated by applying the GBD2013 DW of 0.441 for the ictal state of migraine [29], was 5.2% (0.441*11.7%).

The corresponding calculation for TTH is also shown in Table 1. Headache was reported on an average of 2.4 ± 2.1 days/month, with a mean intensity of 2.4 ± 0.5 (also moderate to severe pain). Duration was again highly variable (mean 34.9 ± 48.2 h), and >24 h, but since days affected were only 2.4/month we took this to imply a mean attack frequency of 1.5/month. The mean proportion of total time spent in the ictal state was 7.3% (100*[1.5*34.9]/[30*24]%) and the disability level (mean disability attributed to TTH per adult with the disorder) was 0.27% (0.037*7.3%).

For pMOH (Table 1), mean headache frequency was 23.3 ± 6.8 days/month and mean intensity 2.95 ± 0.2 (severe pain; all but one participant reported 3). Mean duration was 18.6 ± 1.5 h (all but five participants reporting 24 h). Mean time in ictal state was 60.2% (100*[23.3*18.6]/[30*24]%) of total time. Disability level (mean disability attributed to pMOH per adult with the disorder) was 13.4% (0.223*60.2%).

For other headache on ≥15 days/month (Table 1), mean reported headache frequency was 24.9 ± 6.8 days/month and mean intensity 2.6 ± 0.6 (moderate to severe). Mean duration was 17.9 ± 8.6 h. Mean time in ictal state was 61.9% (100*[24.9*17.9]/[30*24]%) of total time. In order to calculate disability for this group, for whom we had no definitive diagnoses, we assumed a DW for the ictal state of 0.197, taking the DW for pMOH and adjusting it according to the relative headache intensities (*ie*, 0.223*2.6/2.95). Thus, disability level was 12.2% (0.197*61.9%).

Lost productive time estimates (Table 2), with medians of 0 throughout, indicated that >50% of people with all headache types lost no productive time at all. Highly-disabled minorities accounted for most of these losses.

**Table 2:** Lost productive time in the preceding 3 months, per-person and, for paid work, at population level, by headache type

Headache type	Days lost from paid work			Days lost from household work		Lost leisure occasions
	n	%^a^		n	%^a^	n
		per person	population level			
Migraine (*N* = 452)	2.9 ± 5.9 [0; 0; 4]	4.5	0.80	4.8 ± 8.3 [0, 0, 8]	5.3	0.8 ± 1.7 [0; 0; 1]
Tension-type headache (*N* = 493)	1.3 ± 3.0 [0; 0; 2]	2.0	0.41	1.1 ± 3.4 [0; 0; 0]	1.2	0.2 ± 0.5 [0; 0; 0]
Probable medication-overuse headache (*N* = 21)	19.0 ± 31.9 [0; 0; 30]	29.2	0.20	14.4 ± 23.6 [0; 0; 20]	16.0	4.1 ± 9.1 [0; 0; 3.5]
Other headache on ≥15 days/month (*N* = 67)	5.3 ± 11.7 [0; 0; 4]	8.2	0.22	9.7 ± 21.0 [0; 0; 6]	10.8	2.5 ± 11.5 [0; 0; 1]

Values of n are means ± SD [quartile 1; median; quartile 3]. ^a^Percentage of total days available for paid work (denominator 65) or household work (denominator 90).

On average, people with migraine lost 2.9 days from paid work in the preceding 3 months, and more, 4.8 days, from household work. Assuming a 13-week period had 65 workdays, we calculated a loss of 4.5% of paid workdays. For household work we took a denominator of 90 days, giving a loss of 5.3%. These numbers disguised large gender differences associated with working practices in Ethiopia: males with migraine lost 4.9 ± 7.0 days from paid work and 2.2 ± 6.1 days from household work, females 1.9 ± 5.0 (*p* < 0.0001; t-test, 2-sided) and 6.1 ± 8.9 (*p* < 0.0001) respectively.

People with TTH lost relatively little time (≤2.0%) (Table 2).

Those with pMOH lost heavily: from paid work, 19.0 days (29.2%) and from household work 14.4 (16.0%) (Table 2). Here again we observed large gender differences but, with small numbers, only the difference in household work was significant: males (*n* = 6) lost 36.3 ± 43.6 days from paid work and 3.0 ± 7.3 days from household work, females (*n* = 15) lost 12.1 ± 24.4 (*p* = 0.246) and 19.0 ± 26.4 (*p* = 0.0482) respectively.

Losses from other headache on ≥15 days/month were also quite substantial: 8.2% from paid work, 10.8% from household work (Table 2).

### Quality of life

Data on QoL were collected from all participants, whether reporting headache or not (Table 3). Mean summed score was 30.5 (possible range, 8–40) for no headache. All headache types were associated with significant reductions, the greatest being for pMOH and other headache on ≥15 days/month.

**Table 3:** Quality of life and willingness to pay for effective headache care, by headache type

Headache type	Quality of life (WHOQoL-8 summed score) mean ± SD	Willingness to pay		
		N^a^	Absolute (USD/month) mean ± SD [median]	% of income mean ± SD [median]
No headache (*N* = 1314	30.5 ± 3.1	-		
Migraine (*N* = 452)	29.5 ± 3.0 (*p* < 0.0001)	406	1.57 ± 1.91 [0.79]	8.5 ± 18.5 [3.0]
Tension-type headache (*N* = 493)	29.3 ± 3.0 (*p* < 0.0001)	473	1.32 ± 1.74 [0.79]	3.9 ± 6.2 [2.5]
Probable MOH (*N* = 21)	26.3 ± 4.6 (*p* = 0.0005)	11	1.54 ± 1.97 [1.06]	2.1 ± 1.8 [1.3]
Other headache on ≥15 d/m (*N* = 67)	27.0 ± 3.4 (*p* < 0.0001)	64	1.46 ± 1.34 [1.06]	4.6 ± 5.1 [2.4]

^a^Not all participants answered the questions on WTP, or on income levels. *MOH* medication-overuse headache, *d/m* days/month. Values of *p* are for comparisons with no headache (Student’s t-test, 2-sided)

### Willingness to pay

Income levels – < USD 1.00 per day for 40% of participants [13] – reflected Ethiopia’s status as a low-income country, and WTP in turn reflected this. In absolute terms, WTP varied little between headache types: from USD 1.57 per month for migraine down to USD 1.32 for TTH (Table 3), in all cases slightly more among males than females (overall *p* < 0.0001 [Wilcoxon]; data not shown). As percentage of income, mean WTP varied more widely, but, since income distributions were very highly skewed, medians were more indicative (Table 3). The difference between migraine (3.0%) and TTH (2.5%) was significant (*p* = 0.0061 [Wilcoxon]). Participants with pMOH would pay the least proportion of income, according to both mean and median, but these estimates were based on 11 participants only (Table 3).

### Population-level burden

Returning to Tables 1 and 2, we made estimates of burden at population level, and on society. Each person with migraine was, on average, 5.2% disabled, while the prevalence of migraine among 18–65 year-olds was 17.7%. Thus there was 0.92% (5.2*0.177) disability attributable to migraine among the entire population of this age. Those with migraine lost 4.5% of paid workdays, which diluted to 0.80% among this population.

For TTH, with individual disability of 0.27% and a prevalence of 20.6%, population disability was 0.06% (Table 1). Lost paid workdays of 2.0% (Table 2) diluted to 0.41% among the population of 18–65 year-olds. For pMOH, individual disability of 13.4% and a prevalence of 0.7% gave rise to population-level disability of 0.09% (Table 1). Lost paid workdays of 29.2% (Table 2) diluted to a population level of 0.20%. For other headache on ≥15 days/month, individual disability of 12.2% and a prevalence of 2.5% [13] resulted in population-level disability of 0.30% (Table 1), while lost paid workdays of 8.2% diluted to 0.22%.

In total, population-level disability was 1.4% and lost paid workdays were 1.6%.

### Headache yesterday

Headache frequency overall (including unclassified headache) was 4.6 ± 2.7 days/month. Thus, for the 44.9% of participants with any headache, the average probability of headache on any particular day was 0.15 (4.6/30). The predicted 1-day prevalence of headache was therefore 6.9% (0.15*0.449*100), consistent with the observed 7.1% prevalence of HY reported in the sample [13].

We enquired into effect of HY on activities yesterday. Of 169 responders to the question, only 17 (10.1%) reported that they had been able to do everything as normal; 79 (46.7%) claimed they could do less than half or nothing at all (60 of 124 females [48.4%], 19 of 45 males [42.2%]; chi-squared = 0.5041; *p* = 0.478). We took 46.7% as the average lost “output” per person with HY; this deficit, spread among the population according to the gender- and habitation-adjusted prevalence of HY of 6.4%, would be 3.0%.

## Discussion

Our earlier manuscript showed headache to be as prevalent in Ethiopia as elsewhere in the world [13]: migraine was in fact more common (1-year prevalence 17.7%) than the global estimate from GBD2010 (14.7%), and TTH as common (20.6% versus GBD’s 20.8%) [16]. Here, estimating the levels of burden arising from the key headache disorders, we find these to be commensurately high.

In summary, symptom burdens affect, particularly, those with migraine or pMOH. The former, we estimated, spent on average 11.7% of all their time in the ictal state, with headache described as moderate to severe (2.6 on the scale 1–3). The consequent disability burden averaged over time was 5.2%. People with pMOH are only 0.7% of the adult population but, as might be expected, carry much more individual disability: 60.2% of their time was spent in the ictal state, a huge loss of healthy time, with headache rated severe (2.95 on the scale 1–3) and a disability burden of 13.4%. Disability from TTH was much lower. Consequential losses of productive time due to migraine were 4.5% from paid work and 5.3% from household work, and, again as expected, much higher at 29.2 and 16.0% from pMOH. At population level (among those aged 18–65 years), we estimated disability from migraine at 0.92%, with 0.80% of paid workdays lost, from pMOH at 0.09%, with 0.20% of paid workdays lost, and from other headache on ≥15 days/month at 0.30%, with 0.22% of paid workdays lost. Total population-level disability was 1.4% and lost paid workdays slightly higher at 1.6%.

These estimates were not all reflected exactly in other measures of burden. The losses of productive time to migraine (4.5% from paid work and 5.3% from household work) days were consistent with the estimated underlying disability level of 5.2%. Productive-time losses to TTH, while smaller (2.0% and 1.2% respectively), nevertheless substantially exceeded the estimated 0.27% disability. This might be explained by the skewed distributions, and a relatively highly-disabled minority with frequent headache; alternatively it suggests the DW of 0.037 attributed to TTH in GBD2013 [29] is too low. The losses for people with pMOH (29.2 and 16.0%) were also greater than the estimated disability level of 13.4% (the former more than double).

Corroboration of these estimates came from enquiry into HY. This enquiry obviates the potential problem of faulty recall over periods of 3 months [14]. In fact, the predicted 1-day prevalence of headache based on reported frequency over the preceding 3 months was 6.9% - very close to the 7.1% reported prevalence of HY [13]. As for the effect of HY on activities yesterday, the lost “output” per person in the entire population of 3.0% was entirely compatible with the averaged lost paid workdays of 1.6%, given that the former included all planned activities (additionally household work, which had similar losses to paid work, and social events).

The obvious comparison to make is with Zambia, where LTB conducted a similar study with the same methodology and questionnaire [10, 30]. Both Ethiopia and Zambia are landlocked but ethnically and ecologically diverse African countries. Ethiopia, in East Africa, is <20% urbanised, with agriculture accounting for some 85% of the labour force [31], but its capital, Addis Ababa, sits at 2400 m. It is a low-income country: despite a rapidly-growing economy, its GDP per capita remains one of the lowest in the world, and only 4.7% of this is spent on health (latest estimate from 2011 [32]). Many of the rural population in particular live in poverty. Its recent history is troubled, including foreign occupation, war (both civil, and with neighbouring Eritrea), genocide and drought. Zambia, much further south, is one of the most highly urbanised countries in SSA (>40%), with sparsely populated rural areas [33]. Nevertheless, agriculture provides most jobs, although the country’s economy is dependent on copper-mining and vulnerable to fluctuations in copper pricing. It is a lower-middle-income country: its per-capita GDP is 50% higher than Ethiopia’s, with 6.1% spent on health (latest estimate from 2011 [34]); nonetheless, >60% of Zambians live below the poverty line (latest estimate from 2010 [35]). It is politically stable.

The two countries therefore have many differences that might affect the prevalence and impact of headache disorders. In fact, in terms of prevalence, there is slightly less migraine (17.7%) in Ethiopia than in Zambia (22.9%; chi-squared = 12.7602; *p* = 0.0004), a similar level of TTH (20.6% versus 22.8%; chi-squared = 2.1126; *p* = 0.1460), but *much* less pMOH (0.7% versus 7.1%; chi-squared = 115.319; *p* < 0.0001) [13, 30]. GBD2015 refuted any notion that headache disorders are associated with poverty [18, 36]. Rather, in both these countries, pMOH is associated with higher income (ORs of 2.1 in Ethiopia [13] and 6.0 in Zambia [30]). The relative poverty of Ethiopia might, therefore, be one explanation of the difference in prevalence of this disorder – quite simply, Ethiopians cannot afford to overuse medications. A second and probably more influential factor is the greater urbanisation of Zambia: again in both countries, pMOH is very strongly associated with urban dwelling (ORs of 6.1 [13] and 8.6 [30]). This is likely to be explained by poor rural access to medications.

As for burden, people with migraine in Zambia [10] were 4.3% disabled overall (Ethiopia 5.4%), losing 6.3% of paid workdays (Ethiopia 4.5%) and 4.2% of household workdays (Ethiopia 5.3%). These are not highly dissimilar findings. In Zambia but not Ethiopia, lost paid worktime exceeded the underlying estimated disability level, which again may reflect the poverty of Ethiopia (people cannot afford to miss paid work). In Zambia, estimated disability from migraine in the entire working population was 0.98%, in Ethiopia very similar (0.92%); but in Zambia, 1.4% of all workdays were lost to migraine, in Ethiopia only 0.80%. People with pMOH in Zambia were 8.3% disabled and lost 7.4% of paid workdays and 5.0% of household workdays [10]. These estimates are different from those in Ethiopia (13.4%, 29.2% and 16.0%), but, with such different prevalences (7.1% [30] versus 0.7% [13]), and numbers in Ethiopia very small, nothing should be made of this. In other words, with similar prevalences of all but pMOH, the modest differences in headache-attributed burden can be explained by geographical, cultural and environmental differences between these two countries. This suggests that, in the absence of reliable data from elsewhere, the findings in these two countries can reasonably be extrapolated to others in SSA – at least in the east and south.

An important part of our purpose was to inform policy, which the population-level findings do very well. Considering first the study limitations, in our earlier paper we recorded our inability to conduct a diagnostic validation [13]. The diagnostic questionnaire had, however, been employed successfully in multiple other countries and cultures [15]. Furthermore, the essential messages here for policy purposes relate to headache overall rather than to any headache type, and they are not significantly affected by this limitation. The study had several strengths, also noted previously [13]. We employed population-based sampling, included diverse regions with a large sample of >2400, applied ICHD-II diagnostic criteria [26] and used established methodology also tested in numerous other countries [14].

The policy messages are these. Headache disorders are not only common in Ethiopia but also heavily burdensome. Individual disabilities attributable to all headache types make up a total disability of 1.4% among the entire population aged 18–65 years (compared, incidentally, with 1.6% in Zambia [10]). It is important to recognise that this population is effectively the working population. Lost paid workdays in this population from headache total 1.6%, slightly less than the 1.9% in Zambia [10] but substantially more than the 1.1% estimated in a similar LTB study in India [37]. This is an enormous economic burden in a low-income country, likely to be reflected in national productivity and gross domestic product (GDP).

## Conclusions: What is to be done?

At population level, Ethiopia may lose 1.6% of its GDP to headache, but the country has many other health-care problems. Communicable diseases (including HIV) and malnutrition, along with lack of access to clean water for nearly half the population [38], are high among the causes of ill-health. The major non-communicable diseases (cardiovascular disease [CVD], cancer, diabetes and chronic obstructive pulmonary diseases) have been less well documented but nonetheless also contribute substantially to morbidity, with CVD notably on the rise [39]. At the same time, doctors are relatively few [40]. Nevertheless, headache disorders are not only treatable [22] but cost-effectively treatable [23]. As in Zambia [10], WTP would contribute little towards the cost of care, but this should not be a disincentive to finding the solution. Health politicians need to sit down with experts and discuss what must be done to alleviate the headache burden, and how, not just because people in Ethiopia lose much of their health and quality of life to headache but also with the expectation of cost-saving nationally [21]. WHO has recommended structured headache services with their basis in primary care as the most efficient, effective, affordable and equitable solution [21], and the model proposed by LTB for Europe [41], which is highly adaptable, could be reworked to match the health-care infrastructure of Ethiopia.

## Abbreviations

CI: confidence interval
d/m: days/month
DW: disability weight
ETB: Ethiopian birr
GBD: Global Burden of Disease
GDP: gross domestic product
HALT: headache-attributed lost time
HARDSHIP: Headache-Attributed Restriction, Disability, Social Handicap and Impaired Participation questionnaire
ICHD: International Classification of Headache Disorders
LTB: *Lifting The Burden*
MOH: Medication-overuse headache
OR: Odds ratio
pMOH: Probable MOH
SD: Standard deviation
SNNPRS: South Nations Nationalities and People’s Regions States
SSA: Sub-Saharan Africa
TTH: Tension-type headache
USD: United States dollar
WHO: World Health Organization
WTP: Willingness to pay
YLD: Year of life lost to disability
